# An assessment of the move your way program among hispanic adults in Las Vegas, Nevada

**DOI:** 10.1186/s13690-023-01201-4

**Published:** 2023-11-02

**Authors:** Timothy J. Bungum, Jennifer R. Pharr, Courtney A. Coughenour, Maxim Gakh

**Affiliations:** School of Public Health, Department of Environmental and Occupational Health, University of Nevada – Las Vegas, 4700 S. Maryland Parkway, Box 3063, Las Vegas, NV USA

**Keywords:** Physical activity knowledge and behavior, Move your way, Hispanic adults, Cross sectional study, Survey

## Abstract

Physical activity (PA) is important because of its associated health benefits. However, many Americans remain inactive. The 2018 guidelines recognize that PA bouts of less than 10 min are beneficial. The U.S. Department of Health and Human Services’ Office of Disease Prevention and Health Promotion created the Move Your Way® (MYW) campaign to provide PA resources for communities and healthcare providers. This study aims to describe the reach of the MYW campaign, and assess whether having seen, heard, or read (SHR) about MYW, or having seen the MYW logo, was associated with PA knowledge, self-efficacy, and behavior among Hispanics residing in the Las Vegas, Nevada area. Methods: Hispanics, aged 18–74, were surveyed at community events (n = 481) or via an online survey (n = 123). Respondents were asked if they had SHR of MYW, were aware of 2018 PA recommendations, and to report their personal PA behavior, PA knowledge, PA intentions, and demographics. Results: Respondents (71% female) completed a 38-42-item survey. Approximately (12.4%) had SHR of the MYW campaign and 16.1% had seen the MYW logo. Only 3.4% and 15% identified, “150 minutes per week” and “75 minutes per week” as the 2018 guidelines for moderate physical activity (MPA) and vigorous physical activity (VPA), respectively. Those who had SHR of the MYW campaign were more likely to correctly identify 150 min per week as the MPA guideline and had greater confidence in overcoming selected PA barriers. Regression analysis showed that those who had seen the MYW logo were more likely to meet strength guidelines, and that those who had SHR of MYW or saw the logo were more likely to know the recommended minutes of MPA. Conclusions: Knowledge of the MYW program positively associates with some PA behaviors and PA correlates.

**Supplementary Information:**

The online version contains supplementary material available at 10.1186/s13690-023-01201-4.

**Table:** 

Text box 1. Contributions to the literature
• There is limited evidence of the reach of national physical activity (PA) initiatives among adult Hispanics in America.
• Exposure to the Move Your Way program enhances some correlates of PA, and select PA behaviors.
• Additional public health messages that highlight government sanctioned PA recommendations, and PA opportunities that target American Hispanic adults, are needed.

Health-related benefits of regular PA have been recognized since the 1970’s [1]. Such benefits have been demonstrated to occur among people of all ages, abilities, races/ethnicities, and body mass index (BMI) categories. Specific health benefits include improved mental and brain health; assistance with weight management and the prevention of diabetes and some cancers, including breast cancer; strengthening of muscles and bones; improvement in the ability to do everyday activities; and an increased likelihood of engaging in other health behaviors [2–5]. More importantly, regular PA reduces all-cause mortality, coronary artery disease, and hypertension [3, 6, 7].

Professional associations have published PA guidelines specifying the intensity, duration, and frequency that were believed necessary to produce health benefits since the 1970’s [8]. As PA knowledge has advanced, these guidelines have been revised accordingly. For instance, the American College of Sports Medicine (ACSM) published its first PA and testing guidelines in 1978. ACSM recently published its 11th edition of those guidelines [9] The United States (U.S.) federal government waited for more convincing evidence and did not develop and adopt its first set of guidelines until 2008 [10].

To update the 2008 Physical Activity Guidelines and incorporate the most current research and knowledge, a national Physical Activity Guidelines Advisory Committee (PAGAC) was created. The PAGAC developed the scientific report from which the U.S. federal government subsequently created guidelines [11]. These revised guidelines were published in 2018 and are similar to the 2008 version. Both recommend at least 150 min of moderate physical activity (MPA) and a minimum of two muscle-strengthening sessions per week. However, the 2018 guidelines relax some of the rigidity of those articulated in the earlier version. For instance, the 2018 guidelines recognize that PA bouts of any duration produce health benefits, eliminating the 2008 recommendation that bouts be ten minutes or longer [12]. Additional changes presented in the 2018 guidelines include how the recommended durations of MPA and vigorous physical activity (VPA) are expressed. Both the 2008 and 2018 guidelines recommend at least 150 min of MPA spread over the week, rather than the more inflexible 30 + minutes, five or more days per week. A minimum of 75 min per week is recommended for VPA. Either 150 min of MPA, or 75 min of VPA, or an equivalent combination of VPA and MPA, meet the guidelines. The potential applications for these guidelines are apparent. Policymakers and health professionals can use them in their efforts to enhance several aspects of PA at the population level. These include the percentage of children and adolescents who play sports, increasing rates that adolescents and adults walk or bike for transportation purposes, and reducing the prevalence of adults who do not engage in PA [12].

Despite the abundance of literature touting the health benefits of PA, most American adults are not physically active at recommended levels. Less than 25% of men, women, and teenagers meet current guidelines for both aerobic and muscle strengthening PA [12]. These rates are even lower among People of Color, including Hispanics [13], though research shows variation in PA among groups within the Hispanic population [14]. Therefore, PA may be an especially important health behavior for certain Hispanic sub-groups. This is because in the U.S., diabetes and heart disease are two of the leading causes of death among Hispanics [15].

Because of the importance of PA, it is not surprising that for decades researchers have attempted to determine why some people are physically active and others are not [16]. Such research has identified personal and environmental factors that correlate with PA behavior. While several PA correlates have been identified, lack of awareness about guidelines could contribute to low PA levels. In fact, recent research showed that only 36% of American adults were aware that federal guidelines exist. Further, only 1% knew that 150 min of MPA per week was recommended, and 10% knew that PA guidelines for youth exist [17].

To bridge this knowledge gap, the Office of Disease Prevention and Health Promotion (ODPHP) which is an office under the auspices of the U.S. Department of Health and Human Services, developed the Move Your Way® (MYW) campaign. The purpose of the MYW campaign is to enhance knowledge of PA guidelines and increase PA among Americans. MYW is a multi-level community-based communication campaign. Its website hosts PA information and resources for individuals, communities, and healthcare providers. The website provides access to campaign materials and resources, a community playbook, and a partner promotion toolkit to aid implementation of the campaign at the local level [18].

In early 2021, the Southern Nevada Health District (SNHD) was contracted by Communicate Health, a partner of the ODPHP, to deliver local programming of the MYW campaign to the area’s Hispanic population. Hispanic adults were an important population for this intervention because physical activity rates among them are relatively low [19] and this population has been difficult to study [20]. Third, Hispanic adults are overburdened by some PA related chronic health conditions, such as overweight and obesity, and diabetes [21]. Finally, Hispanics comprise a large proportion of residents in the Las Vegas, Nevada metropolitan area, at about 33% [22].

The MYW campaign’s products and messages are evidence-based. Evaluating PA interventions can help inform decisions about programs, policy, and funding and improve practice. However, evaluating PA interventions can present methodological challenges and there is yet no clear consensus in research or practice about which of the over 71 evaluation frameworks are appropriate to evaluate PA interventions. As a result, we built our outcome-focused evaluation around the Transtheoretical Model because MYW is grounded in the Transtheoretical Model [23] and is designed to impact the behavior of PA contemplators (those who are not meeting the guidelines, but plan to be more physically active in the next six months). Our goal was to understand the sociodemographic factors, barriers, motivators, and preferences that are affected by knowledge and the awareness of PA guidelines among PA contemplators. This study aimed to assess the reach of MYW and the relationship between having seen, heard, read (SHR) about the MYW campaign, or having seen the MYW logo, with knowledge of current MPA and VPA guidelines among Hispanic adults in Las Vegas. We also assessed the relationship of having SHR and seen the logo with muscle-strengthening activities guidelines, current PA behavior, and intention to increase PA in the next six months.

## Methods

### Study design and setting

Several strategies were used to deliver the MYW program in Las Vegas. Through Communicate Health, the ODPHP paid for social media that promoted the MYW campaign among Hispanic audiences in the community. During the spring, summer, and early fall of 2021, the SNHD organized and delivered community-based intervention programs. Numerous strategies were employed that included the distribution of MYW materials such as PA fact sheets and pamphlets, and hosted MYW events in predominantly Hispanic neighborhoods that provided opportunities to engage in PA and learn about MYW. MYW materials articulated the 2018 PA guidelines (150 min/week of MPA or 75 min of VPA per week and muscle-strengthening activities performed at least twice weekly). Materials were available in Spanish and English and were disseminated in person at events by SNHD staff. MYW materials were also distributed at other events, including COVID-19 vaccination clinics, PA classes, a YM/YWCA dance party, a virtual 5-kilometer footrace, and an online Girls on the Run event. The SNHD website also houses a link to MYW campaign information [22].

### Data collection

Upon completion of the MYW intervention events in mid-September 2021, our research team commenced data collection that assessed the PA knowledge, intentions, and behaviors of the target population. The Framework for Outcome Assessment [24], sometimes used in physical activity knowledge and behavior outcome evaluation, proports that using a survey to assess self-reported health outcomes and related knowledge or attitudes is acceptable. Much of the data collection occurred at community events, 13 of which were described as “resource fair/vaccination clinics,” where attendees could receive COVID-19 vaccinations and a range of health-related materials and services. Skin care, vision and hearing tests, body fat assessment, and other services were provided. Data were also collected at two community celebrations and two free food distributions. At all events, those who approached our booth/table were asked if they were interested in completing a PA knowledge and behavior survey. Prospective participants were also informed that upon completion of the survey, which consumed 5–7 min, they would receive a $5 Walmart gift card. All data collections were held in geographic areas where at least 50% of residents identified as Hispanic. Data collection was completed in early March 2022. The project was approved by the university’s Office of Research Integrity (Protocol 1772545-1).

At all survey sites, at least one student and a university faculty member were situated at a table adorned with a banner announcing our university affiliation and a sign explaining that we were conducting a study addressing exercise/PA knowledge and behavior. Those who agreed to participate used their phones to access the survey via a QR code. The survey was available in English and Spanish. The English version had been translated into Spanish and back-translated to ensure accuracy and cultural relevance. Minor differences were discussed, and a final version was agreed upon. Respondents without access to smart phones (< 15%) were given the option to take the survey using available iPads.

### Study population

Inclusion criteria were that participants were between 18 and 74 years of age, self-identified as Hispanic, and reported living in the Las Vegas Metropolitan area. Since the survey was available in both English and Spanish, participants were instructed to select the survey language with which they were most comfortable. We additionally collected 123 online surveys via the data collection agency, Qualtrics (Provo, UT) [25]. These surveys were completed in English over a two-week period in late February.

The survey consisted of 38–42 items, depending on responses to specific items and their associated skip-logic patterns. Demographic data were gathered. Variables related to the MYW campaign exposure were gleaned from questions regarding whether participants had: (1) SHR anything about the MYW campaign in the past six months; (2) seen the MYW logo over the same time period, and (3) SHR anything about federally-created PA guidelines from any source. Frequency and duration of being physically active was measured by asking, “In a typical week, how many days do you do any aerobic physical activity of at least moderate intensity, such as brisk walking, bicycling, using a cardio machine, or swimming?” and, “On the days that you do any physical activity of at least moderate intensity, how long (in minutes) do you typically do these activities?” The same questions were used for VPA. The following equation was employed to determine if the aerobic guideline had been met, (minutes VPA x 2) + minutes MPA ≥ 150).

Frequency of muscle-strengthening exercise was measured by asking, “In a typical week, how many days do you do physical activities specifically designed to strengthen your muscles, such as lifting weights or body weight exercises (like push-ups)?” To measure intention to become more physically active, respondents were asked, “How likely are you to become more physically active in the next six months?” Response choices were, “extremely unlikely, somewhat unlikely, somewhat likely, extremely likely.” For analysis, “extremely unlikely” and “unlikely” were combined into “unlikely.” The same grouping strategy was used for the “extremely likely” and “likely” responses. Those who responded “somewhat” or “extremely” likely were designated as PA “contemplators.” There were also eight items presented on a 1–10 scale that measured respondents’ confidence (self-efficacy) that they could overcome PA barriers (bad weather, bored by PA, PA-related pain, exercising alone, feeling tired, busy with other activities, not enjoying PA, feeling depressed) and still remain physically active.

Participants were also asked questions involving knowledge typical of physiological responses to vigorous and moderate intensity physical activities. Correct responses to these items were, “your heart beats much faster than usual and you cannot say more than a few words before stopping for a breath,” and “your heart beats somewhat faster than usual and you can talk while doing the activity,” respectively. Knowledge of the 2018 PA guidelines was measured by the following multiple- choice items: (1) “What duration of moderately intense PA is recommended?” Correct answer: 150 min per week. (2) “How many days of muscle-strengthening activities are needed per week to gain strength?” Correct answer: a minimum of two. (3) In addition, a review of our original survey revealed that we had failed to include a question regarding the recommended minutes of VPA. The following item was a part of the Qualtrics on-line survey, “What duration of vigorous PA is needed to produce health benefits?” Correct answer: 75 min per week.

### Statistical analysis

Frequencies and distributions were calculated for all variables. Binary, univariate logistic regression was used to calculate odds ratios (OR) and binary, multivariate logistic regression was used to calculate adjusted odds ratios (AOR) for exposure to the MYW campaign or MYW logo and PA behaviors, intention, and knowledge. Cross-tabulations were used to conduct Pearson chi square analyses to assess relationships between exposure to the MYW campaign and PA knowledge and behaviors. One-tailed, independent t-tests were used to determine if exposure to the MYW campaign or if having seen the MYW logo were associated with confidence in overcoming barriers to PA. If data for a dependent variable were missing, then those participants were omitted from the data analysis for that particular variable, but were retained for other analyses. This causes some analyses to use slightly different sample sizes. In all cases, alpha was set at 0.05 to determine statistical significance. SPSS version 28.0 was used for all analyses [26].

## Results

The survey was completed by 610 Hispanic adults. Most surveys were collected in person (n = 487) and 123 were completed online. A majority of respondents (70.6%) were female and the overall mean age was 37(11.7) years. A plurality (36.1%) of participants were high school graduates/GED recipients, while 22.1% had attended some college. In general, annual incomes were low, with nearly 30% earning below $20,000. Approximately one-half of respondents completed the survey in Spanish. Full demographic information is presented in Table 1.

**Table 1:** Demographic and Physical Activity Characteristics of a Sample of Surveyed Hispanic Adults Residing in the Las Vegas, Nevada Metropolitan Area (n = 610)

Variable	n (%)
Gender	
Female	424 (69.5)
Male Missing	139 (24.2) 47 (6.5)
Education	
Less than high school	75 (12.3)
High school graduate / GED	220 (36.1)
Some college	135 (22.1)
Associate degree	40 (6.6)
Bachelor’s degree	73 (12.0)
Graduate degree	25 (4.1)
Missing	42 (6.9)
Annual Household Income (K = thousand)	
< 20 K	179 (29.3)
20-34.9 K	161 (26.4)
35-49.9 K	96 (15.7)
50-74.9 K	79 (13.0)
> 74.9 K	50 (8.1)
Missing	45 (7.4)
Race	
White	286 (46.9)
Black or African American	5 (0.8)
American Indian or Alaska Native	13 (2.1)
Asian	3 (0.5)
Native Hawaiian or Other Pacific Islander	5 (0.8)
Two or more	47 (7.7)
Other	203 (33.3)
Missing	48 (7.9)
Parent of child < 18 years old	
Yes	365 (59.8)
No	204 (33.4)
Missing	41 (6.7)
Survey language	
English	304 (49.8)
Spanish	306 (50.2)
Residence	
City	441 (72.3)
Suburb	99 (16.2)
Rural Missing	29 (4.8) 41 (6.7)

There were a few differences in demographic characteristics between contemplators (those who were likely or extremely likely to become more physically active in the next six months) who had SHR about MYW versus contemplators who had not SHR about MYW based on chi square tests. Contemplators who had SHR about MYW were significantly more likely to live in the suburbs (p = 0.02), to have some college or a college degree (p = 0.05), and to be in a higher income (greater than $50,000) bracket (p = 0.03) than those who had not SHR about MYW.

Table 2 displays responses to PA knowledge, PA behavior, and intention to increase PA over the next six months. About 1/8 of respondents had SHR about the MYW campaign, 20.2% had seen the logo, and nearly 60% indicated that they were aware that federal guidelines for PA exist. Only 3.0% correctly answered that the recommended amount of moderate PA was 150 min per week; however, if the correct responses were expanded to include another of their response choices, “30 minutes, five times per week,” the proportion of correct responses increased to 31%. Of the subsample (n = 123) that was asked about the minutes of recommended VPA per week, 15% responded correctly (75 min per week). 18% of the entire sample answered the number of days recommended for muscular strengthening exercise correctly (2 days). Chi square tests revealed that the subsample who answered the survey online were more likely to have higher education (p < 0.01), report a higher income (p < 0.01), and live in the suburbs versus the city (p < 0.01). However, they were not more likely to answer the moderate PA questions correctly (p = 0.25) or the days of strengthening exercise correctly (p = 0.45). Table 2 also shows the prevalence of correct and incorrect response rates to the physiological responses to MPA and VPA. Most correctly answered the item regarding physiological responses to MPA (57%), but 63.6% incorrectly answered the question of physiological response to VPA. We also calculated the rates of those who met the PA recommendations for aerobic and muscle strengthening activity. Our data showed that over 40% of respondents met both the aerobic and muscle strengthening guidelines.

**Table 2:** Responses to Knowledge Questions and Physical Activity Behaviors from a Sample of Hispanic Adults Residing in the Las Vegas Metropolitan Area

Variable		N(%)	
Had Seen, Heard, or Read (SHR) about the MYW Campaign in the Past 6 Months			
Yes		92(15.1)	
No		518(84.9)	
Had Seen the MYW Campaign Logo in the Past 6 Months			
Yes		123(20.2)	
No		487(79.80	
Had Seen, Heard, or Read (SHR) Anything about the Recommended Physical Activity Guidelines^1^			
Yes		349(57.2)	
No		246(40.3)	
Missing		15(2.5)	
Answered Moderate PA: Physiological Response Correctly			
Yes		351(57.5)	
No		237(38.9)	
Missing		22(3.6)	
Answered Vigorous PA: Physiological Response Correctly			
Yes		200(32.8)	
No		388(63.6)	
Missing		22(3.6)	
Answered Minutes/Week Moderate PA Correctly (150 min per week)			
Yes		21(3.4)	
No		561(92.0)	
Missing		28(4.6)	
Answered Minutes/Week Moderate PA as 30 min, five times per Week			
Yes		178(29.2)	
No		440(88.2)	
Missing		28(4.6)	
Answered Minutes/Week Vigorous PA Correctly (75 min per week)			
Yes		18(15.0)	
No		490(85.0)	
Missing		102 (16.7)	
Answered Number of Days of Muscle Strengthening Exercise Correctly (two days)			
Yes		112(18.4)	
No		470(77.0)	
Missing		28(4.6)	
Met the Aerobic Exercise Guidelines			
Yes		255(41.8)	
No		250(41.0)	
Missing		105(17.2)	
Met Muscle Strengthening Exercise Guidelines			
Yes		338(55.4)	
No		261(42.8)	
Missing		11(1.8)	

Intention, more active next 6 months Likely^*^ 259(43.4)

unlikely^**^ 337(56.5) Missing 14(2.3)

^*^extremely likely, or likely; ^**^ extremely unlikely or unlikely

^1^Respondents were asked about US government guidelines, we do not know if they assumed that those guidelines created by professional associations were originated from the federal government

Variables that were significant in the univariate analysis remained significant after controlling for demographic characteristics. Adjusted odds ratios are presented in Table 3. Logistic regression revealed that those who had SHR about the MYW campaign or had seen the MYW logo were no more likely to meet aerobic guidelines than were those who were not exposed to the MYW campaign. The exposed were also no more apt to know the typical physiological responses to VPA or MPA, or know the number of days of muscle strengthening was needed to “make big health gains.”

**Table 3:** Adjusted Odds Ratios for Exposure to the Move Your Way Campaign or its Logo and Physical Activity Behavior, Intention, and Knowledge from a Sample of Hispanic Adults Residing in the Las Vegas, Nevada Metropolitan Area using Multivariate Logistic Regression Model

	SHR about MYW (yes)		Seen MYW Logo (yes)	
	AOR, [95%CI]	P-value	AOR, [95%CI]	P-value
Met Aerobic PA Guidelines (yes)	0.858, [0.509–1.446]	0.565	1.025, [0.638–1.649]	0.918
Met Muscle Strengthening PA Guidelines (yes)	**1.266, [0.761–2.106]**	**0.363**	**1.869, [1.171–2.983]**	**0.009**
Moderate PA Physiological Response Correct (yes)	0.697, [0.412–1.178]	0.177	1.050, [0.655–1.683]	0.838
Vigorous PA Physiological Response Correct (yes)	1.301, [0.760–2.225]	0.337	0.941, [0.575–1.541]	0.810
Moderate PA Minutes Correct (yes)	**4.748, [1.670-13.495]**	**0.003**	**3.526, [1.269–9.798]**	**0.016**
Muscle Strengthening PA Days Correct (yes)	1.580, [0.887–2.814]	0.121	1.090, [0.637–1.865]	0.753
Likely to increase PA in Next 6 Months (yes)	0.938, [0.131–6.742]	0.938	1.285, [0.801–2.062]	0.298

SHR = Seen, heard, or read; MYW = Move Your Way Campaign; AOR = adjusted odds ratio; CI = confidence interval; PA = physical activity

Refence variable = answering “no” to SHR about MYW, seeing the MYW logo, meeting guidelines, and likely to increase PA in next six months and answering incorrectly questions about the contents of the guidelines

Controlling for Age, gender, residence, race, education, income, children, language

Bold = p-value < 0.05

However, participants who had seen the MYW logo were almost two times more likely to have met the guidelines for muscle strengthening (AOR = 1.896, p < 0.05) than those who had not seen the MYW logo. Additionally, those exposed to the MYW campaign had higher PA knowledge for select items. Both those who reported having SHR about the MYW campaign or having seen the MYW logo were over three times as likely to answer the question about the guideline minutes of MPA correctly (AOR = 4.748; p < 0.05 AOR = 3.526, p < 0.05) respectively).

Among contemplators (n = 259), who were 42.4% (259/610) of our sample, having SRH about MYW or having seen the MYW logo impacted outcome variables. Chi square tests revealed that contemplators who had SRH about MYW were significantly (p < 0.01) more likely to have gotten the recommended MPA duration question correct when compared with contemplators who had not SRH about MYW (16.2% vs. 2.7%). Additionally, contemplators who had SRH about MYW were significantly (p = 0.01) more likely to have gotten the muscular strengthening exercise questions correct when compared with contemplators who had not SRH about MYW (35.1% vs. 16.4%). And, contemplators who had seen the MYW logo were significantly (p = 0.03) more likely to have correctly responded to the “amount of moderate exercise” when compared with contemplators who had not (10% vs. 3%). There were, however, no other significant findings (e.g., meeting aerobic and strength guidelines, knowing the correct physiological responses, or overcoming barriers to PA).

The relationships of having SHR MYW materials or seen the MYW logo and respondents’ confidence to overcome barriers to aerobic and muscle-strengthening activities are presented in Tables 4 and 5. Independent t-tests revealed that those who were exposed to the MYW campaign had higher confidence (measured via higher mean confidence scores) for overcoming some barriers to PA: Those who reported having seen the MYW logo had higher confidence that they could be physically active enough to stay healthy if they felt pain while being active (p < 0.05), did not enjoy PA (p = 0.05), or were busy with other activities (p = 0.04).

**Table 4:** Independent t-Tests for Confidence in Overcoming Barriers to Physical Activity based on Having Seen, Heard, or Read about the Move Your Way Campaign in the Past 6 Months from a Sample of Hispanic Adults Residing in the Las Vegas, Nevada Metropolitan Area (n = 610)

How confident are you right now that you could be physically active often enough to stay healthy if:	P-value	Mean Difference	Std. Error Difference	95% Confidence Interval	
The weather was bothering you	0.351	-0.137	0.356	-0.837	0.563
You were bored by the physical activity program or activity	0.331	0.147	0.336	-0.513	0.807
You felt pain when being physically active	**0.048**	**0.556**	**0.334**	**-0.100**	**1.212**
You had to be physically active alone	0.315	-0.176	0.365	-0.893	0.541
You did not enjoy it	0.112	0.433	0.355	-0.264	1.131
You were too busy with other activities	0.162	0.328	0.333	-0.326	0.983
You felt tired	0.223	0.265	0.346	-0.416	0.945
You felt depressed	0.098	0.491	0.380	-0.254	1.237

**Table 5:** Independent t-Tests for Confidence in Overcoming Barriers to Physical Activity based on Having Seen the Move Your Way Logo in the Past 6 Months from a Sample of Hispanic Adults Residing in the Las Vegas, Nevada Metropolitan Area (n = 610)

How confident are you right now that you could be physically active often enough to stay healthy if:	P-value	Mean Difference	Std. Error Difference	95% Confidence Interval	
The weather was bothering you	0.349	0.121	0.312	-0.492	0.734
You were bored by the physical activity program or activity	0.058	0.463	0.294	-0.116	1.041
You felt pain when being physically active	**0.005**	**0.765**	**0.292**	**0.191**	**1.339**
You had to be physically active alone	0.177	-0.297	0.320	-0.926	0.332
You did not enjoy it	**0.005**	**0.810**	**0.310**	**0.201**	**1.420**
You were too busy with other activities	**0.040**	**0.513**	**0.292**	**-0.060**	**1.087**
You felt tired	0.105	0.369	0.293	-0.207	0.944
You felt depressed	0.234	0.245	0.336	-0.416	0.905

## Discussion

Our data shows that a relatively small proportion (15%) of our sample had SHR about the MYW campaign, or had seen the associated logo (20%) over the previous six months. Although the rates appear fairly low, research shows that the benefits of prevention innovations are slow to be recognized and even slower to be adopted [27]. At any rate, the proportion of respondents who had SHR about the MYW campaign was moderate and suggests that the brand recognition of the MYW campaign is low. That being said, the Diffusion of Innovation Theory posits that how an idea or behavior spreads depends on numerous factors in a social system and that some people, populations, or entities adopt the innovation quicker than others [27]. Strategies that may speed up diffusion of a behavior like PA include using entertainment [28] and peer networks [29] to disseminate information about MYW and to encourage peer communication about the 2018 guidelines.

The MYW Campaign is grounded in the transtheoretical model of behavior change and focuses on those in the contemplation stage. Past research has shown that interventions targeted at specified stages are more effective [30]. Thus, the outcome that contemplators were more knowledgeable than the non-contemplators lend credibility to efficacy of the MYW campaign. Contemplators who had SHR of the MYW program were more knowledgeable regarding the updated moderate aerobic guidelines, the recommended frequency of strength activities, and that those who had seen the logo were more likely to have correctly answered the questionnaire item about the amount of MPA needed to produce big health gains than those that had not seen the logo. Although this knowledge was not associated with participation in aerobic or strength activities, it is a step in the right direction because knowledge is believed to the necessary, but not sufficient, to change behavior [31].

Viewing our knowledge data descriptively provides another perspective. Even though those contemplators who had SHR about the MYW program were more apt to know the recommended duration of MPA than contemplators who had not SHR MYW materials, only 16% knew that it was 150 min per week. So even though contemplators who had SHR of MYW were more knowledgeable than were those who had not SHR of the MYW program, few knew the correct guideline. This is however a marked improvement over a similar study completed over a decade ago when less than 1% of Hispanic participants knew the prevailing MPA recommendation [21].

There may be other reasons why small numbers of respondents were able to correctly identify current aerobic guidelines. As stated earlier, the campaign is new and parts of the intervention and data collection were completed during the most severe pandemic in over 100 years [32]. It is understandable that the attention that many people devoted to health issues might have been focused on COVID-19. The pandemic also likely influenced PA behavior, as research shows that at least at the start of the pandemic, PA attenuated among people in the U.S., particularly among Hispanics [33, 34].

Those who had seen the MYW logo were more confident that they could persist in their efforts to be physically active in the face of three barriers. Those specific barriers are experiencing pain, not enjoying PA, and if they were busy with other activities. This is possibly the result of MYW campaign materials that provide specific strategies to overcoming barriers [35]. Though we did not specifically ask about other contextual factors, such as neighborhood barriers, prior research shows that factors such as safety and accessibility may be barriers to PA as well [36]. Another study specifically examining adult Hispanics [37] shows that safety is an issue for those who want to be physically active. Additionally, barriers may be different for certain subpopulations not explored in this study, such as single parent households. Researchers may choose to examine such barriers to develop more targeted interventions.

Greater knowledge among those who had SHR about the MYW campaign were apparent for additional items. A slightly higher percentage of the subsample that was asked about VPA correctly answered that 75 min of vigorous PA per week is now recommended (15%) than was noted in previous work [17]. Because this data was collected late in the study, it may indicate that knowledge levels of the 2018 guideline specifics are slowly climbing. It could also be a consequence of the online sample having a higher educational attainment than those who completed the survey in person. However, while the subsample who answered the survey online were more likely to have higher education, report a higher income, and live in the suburbs versus the city, they were not more likely to answer the moderate PA questions correctly or the days of strengthening exercise question correctly.

As with all research, there are limitations. It would have been ideal to collect data over a shorter time period and sooner after the completion of the intervention. Additionally, our survey used self-reported data, which is subject to recall and social desirability biases. However, names were not attached to the survey, which would minimize the social desirability bias. It is possible that respondents over-reported their PA behavior. But this appears to be unlikely because our data is similar to that from recent national data [38]. In addition, this was a cross-sectional study and therefore causation cannot be determined. While surveys like this are an acceptable method of assessment some frameworks for outcome assessment recommend triangulation of quantitative and qualitative data to better understand outcomes, PA behavior, and knowledge [39].

The addition of qualitative data collection would also aid in better understanding barriers to PA initiation, like contextual factors such as neighborhood safety or lack of facilities. Also, our study includes only a sample of Hispanic participants from the Las Vegas metropolitan area, so findings cannot be generalized to other ethnicities and geographic regions. Relatedly, there is heterogeneity within the Hispanic population that is not accounted for. Furthermore, females were an over-represented portion of our sample, and our sample was more white (46.9% vs. 17.6% in Clark County, NV) and less “other” (33.3% vs. 47.8% in Clark County, NV) and 2 or more races (7.7% vs. 29.7% in Clark County, NV) [40]. Another factor to consider in generalizability is that our sample was recruited from community events, and many of those were health focused events. It is possible that participants are more health conscience than those not attending such events.

## Conclusions

The MYW campaign appears to have had a modest impact on PA knowledge, self-efficacy, and behavior among Hispanics living in the Las Vegas, NV metropolitan area. Exposure to the MYW programs is correlated with some positive outcomes.

Funding was provided by the U.S. Department of Health and Human Services through the and Office of Disease Prevention and Health Promotion.

### Electronic supplementary material

Below is the link to the electronic supplementary material.

Supplementary Material 1

## Data Availability

contact Timothy Bungum at tim.bungum@unlv.edu.
