# Supplementary material for: An assessment of the move your way program among hispanic adults in Las Vegas, Nevada
Source: Arch Public Health. 2023 Nov 2;81:192. doi: 10.1186/s13690-023-01201-4 (PMC10621095; doi:10.1186/s13690-023-01201-4)
Supplement: Supplementary file 1 — Supplementary Material 1 [file 13690_2023_1201_MOESM1_ESM.docx]

Letter to the Editor-Aims and Scope Statement

What is known:

Physical Activity (PA) is an important health behavior. Despite the relationship of PA and healthy being well-established, most Americans are not active at levels that are believed to produce health benefits. Low PA levels have been shown to be prevalent among minority groups, including Hispanics. This deficit in PA could partially be the result of people not knowing the current guidelines. Additionally, in the past, guidelines have been written by academicians for academicians and were not particularly user friendly for community use.

What this study adds:

With the above in mind the Office of Disease Prevention and Health Promotion created the Move Your Way Campaign (MYW) which was designed to make the guidelines easily understood by all audiences. The timing of the MYW initiative is also important because subtle changes were made to the 2008 guidelines that are reflected in the new version which was published in 2018. These changes largely targeted how one’s duration of PA participation is calculated. The seminal difference is that PA bouts no longer need to be of ten minutes duration or longer to be counted towards meeting guidelines. Questionnaire items were written so that knowledge of the subtle changes made to the guidelines can be detected. Because little was known about Hispanics knowledge of the new guidelines, or whether a MYW initiative had penetrated that community in Las Vegas the Office of Disease Prevention and Health Promotion funded an evaluation of the program. This manuscript is the result of research done to determine if the guidelines are known by members of the Hispanic community in Las Vegas, and if respondents had seen heard or read (SHR) of the MYW program.

What are the implications for clinical practice, public health and / or research:

This evaluation is the first to have a sufficient sample size to assess MYW’s impact on the Hispanic community. A relatively small proportion of respondents had SHR of the MYW program, the penetration of the MYW program among Las Vegas Hispanics appears to be modest. We found that awareness of the program and knowledge of the new guidelines correlated with PA among some group members after the MYW media campaign had been completed. There was a significant relationship between exposure to the MYW campaign and some PA components, as well as some select psychosocial variables that are predictive of PA. We learned that a fair number of participants had knowledge of the approximate guidelines to PA, but few had knowledge of the new, specific guidelines. Our study design did not allow us to determine if exposure to the program influenced PA behavior. The implication for future research is that it is likely that additional intervention efforts will be necessary to influence a behavior that is as complex as PA among the Hispanic population. Because the physical environment is a key to increasing PA it is possible the expensive changes to that environment would be necessary to produce large changes in PA behavior.
